# Skin color representation in orthopedic literature

**DOI:** 10.1007/s00402-026-06561-4

**Published:** 2026-11-03

**Authors:** Rahul K. Goyal, Akin A. Adio, Hari Nadiminti, Eric M. Black, Adam Z. Khan, John G. Horneff, Brian W. Hill, Joseph A. Abboud

**Affiliations:** 1 Pennsylvania State University, State College, USA; 2 Perelman School of Medicine, University of Pennsylvania, Philadelphia, USA; 3 Department of Dermatology, Summit Health, Berkeley Heights, USA; 4 Department of Orthopaedic Surgery, Summit Health, Florham Park, USA; 5 Department of Orthopedic Surgery, Southern California Permanente Medical Group, Panorama City, USA; 6 Department of Orthopaedic Surgery, University of Pennsylvania, Philadelphia, USA; 7 Palm Beach Orthopaedic Institute, Palm Beach Gardens, USA; 8 Division of Shoulder and Elbow Surgery, Rothman Orthopaedics, Philadelphia, USA

**Keywords:** Skin color, Health equity, Diversity, Artificial intelligence

## Abstract

**Introduction:**

Underrepresentation of skin color in educational materials can undermine diagnostic accuracy and contribute to disparities in orthopedic care for minority populations. While underrepresentation of darker skin types has already been identified in orthopedic literature, this study assessed whether underrepresentation is apparent in open access journals, is uniform across all orthopedic subspecialties, differs between figure types, and has shifted over time as awareness of skin of color disparities has grown.

**Materials and methods:**

40,674 figures were accessed from 14 open-access orthopedic journals and assigned a subspecialty and a country of origin based on author affiliation. Automatic filters narrowed the dataset to 4,809 figures. Each figure was processed by a GPT-4o artificial intelligence (AI) pipeline to obtain skin presence (yes/no), figure type (clinical, intraoperative, illustration), Fitzpatrick skin type (I-VI), and a confidence score (1–5). Agreement was high with human review for skin presence and figure type and moderate with a board-certified dermatologist for skin tone, with disagreement biased toward darker ratings. 2,025 skin-containing figures were analyzed.

**Results:**

Among 2,025 figures, most depicted light skin (65.6%, Fitzpatrick I-II), 29.6% medium (III-IV), and only 4.8% dark (V-VI). This demonstrated severe overrepresentation of light skin (*p* < 0.001) and severe underrepresentation of dark skin (*p* < 0.001) relative to both a diverse-population reference and US population estimates. Underrepresentation of dark skin was consistent across subspecialties and figure types. Although subgroup analysis revealed modest improvement in dark-skin representation from 2015 to 2024 in non-U.S. figures (r = + 0.72, *p* = 0.02), the year-by-region interaction was not significant (*p* = 0.24).

**Conclusions:**

Within open-access orthopedic literature, dark skin is consistently underrepresented and falls short of US and diverse-population benchmarks, regardless of figure type or subspecialty. This systemic pattern narrows the implicit norm of who an orthopedic patient appears to be and warrants editorial attention given documented disparities in orthopedic care.

**Supplementary Information:**

The online version contains supplementary material available at https://doi.org/10.1007/s00402-026-06561-4.

## Introduction

Concerns regarding skin of color representation in medicine have increased over the past several years, likely attributable to the growing attention given to structural inequities in medicine and the increasingly diverse patient populations of the United States (US) [1]. Stemming from these concerns, recent studies have demonstrated that significant underrepresentation of darker skin types exists in medical education, spanning dermatology, emergency medicine, and general medical training [2, 3]. As a whole, medical textbooks and online teaching resources have been shown to skew toward lighter skin tones [4, 5]. Since clinically important findings, such as bruising, inflammation, and non-blanching erythema, can present differently across different skin tones, underrepresentation of skin color in educational materials can undermine diagnostic accuracy and contribute to disparities in care, especially for minority populations [6–8]. Surveys of medical trainees reinforce this, with most reporting that their resources feature predominantly light skin and that they feel underprepared to recognize disease in patients of color [9]. Orthopedic surgery is a specialty that is heavily reliant on image-based recognition and physical examination, with surgeons and trainees relying on visual patterns to assess soft tissue integrity, infection, vascular changes, and postoperative healing [10–12]. These visual tasks are performed within a specialty in which racial and ethnic disparities in care are already documented. In a meta-analysis of hip fracture surgery, Black patients had 49% greater odds of surgical delay beyond two days and longer inpatient stays than White patients, and disparities in perioperative outcomes persist when racial and ethnic groups are examined individually rather than aggregated [13, 14]. Representation in the visual record is therefore not a stylistic concern but part of the same problem. Therefore, understanding disparities in skin of color representation in orthopedic literature is of critical importance.

While underrepresentation of darker skin types has already been identified in figures published in orthopedic literature [15], several important dimensions that are typically considered when evaluating representation gaps in medical imagery remain unexplored within the field [1]. This includes (1) whether it persists in open access journals, (2) whether underrepresentation is uniform across all orthopedic subspecialties, (3) whether it differs between figure types, and (4) whether it has shifted over time as awareness of skin of color disparities has grown in more recent years. The purpose of this study was to resolve these open questions and therefore enable a more comprehensive understanding of skin of color representation in orthopedic literature. We hypothesized that medium and dark skin tones would be significantly underrepresented relative to both United States (US) and global populations, and that this underrepresentation would persist across subspecialties, figure types, and across the 10-year study period.

## Methods

### Figure harvesting and sampling

Figures were sourced from PubMed Central using the NCBI E-utilities API. 14 open access, English-language orthopedic journals were sampled across 10 years (2015–2024). All journals publish US and globally authored literature. For each journal-year cell, PubMed Central was queried for open-access articles restricted to clinically illustrated article types, comprising case reports, reviews, clinical, comparative and guideline articles, together with articles whose title or abstract indicated a technique, case report or series, or outcomes study. The verbatim search string is given in Supplementary Table 1. To avoid oversampling any single journal or publication year, a maximum of 75 articles per journal were drawn per year. When a cell returned more than 75 eligible articles, 75 were selected by simple random sampling without replacement using a fixed per-cell seed, and otherwise all articles were included. Sampling covered 140 journal-year cells, of which 71 reached the cap, and returned 6,339 articles. Of these, 5,144 articles contained downloadable figures, from which every figure was taken, leading to a total of 40,674 figures. The 14 journals sampled, with the articles and figures contributed by each, are listed in Supplementary Table 1.

### Automated image filtering

**Fig. 1:**
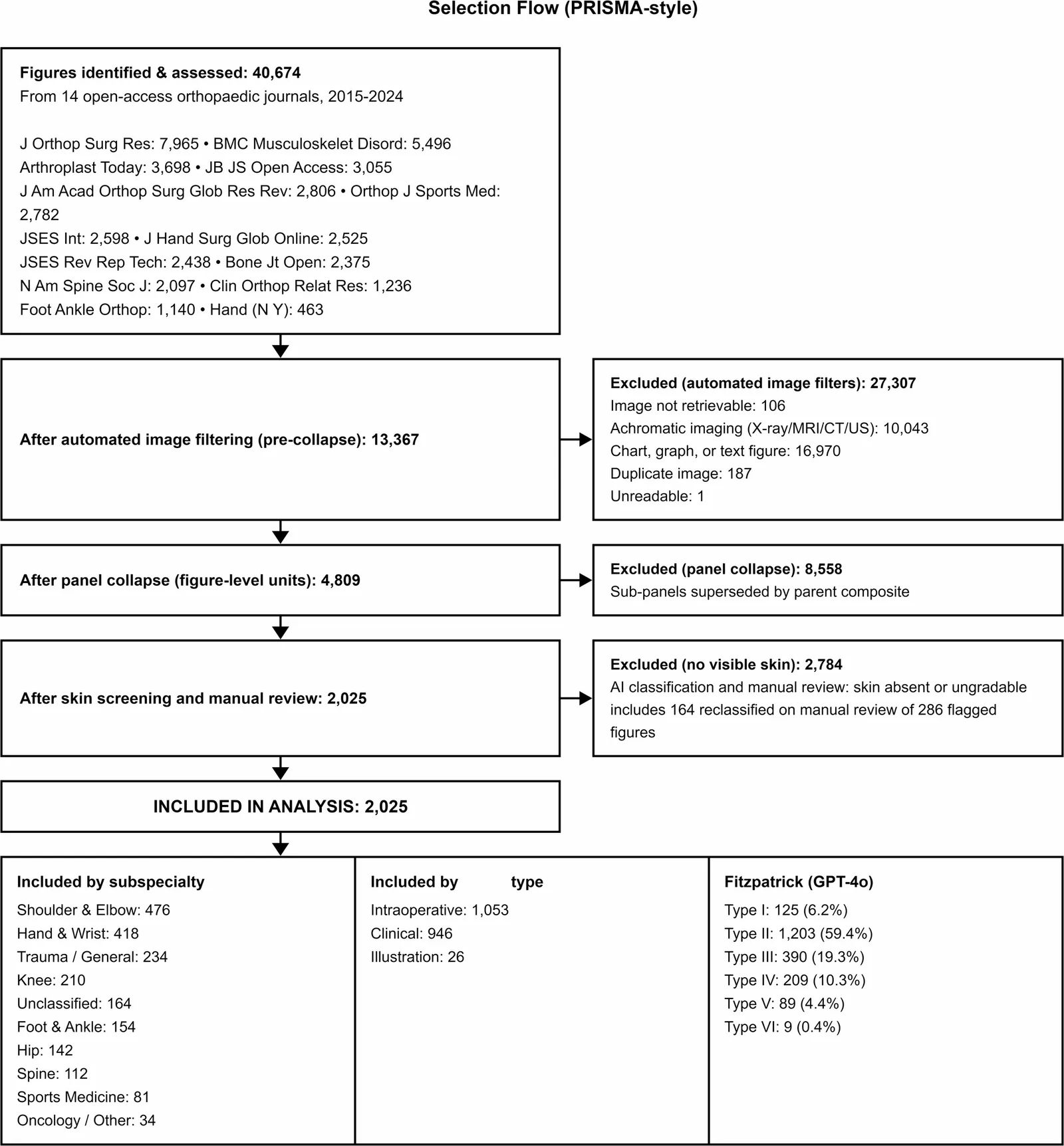
PRISMA-style flow diagram of figure identification, screening, and inclusion. 14 open-access orthopedic journals (2015–2024) were included. The lower panels summarize the 2,025 skin positive figures by subspecialty, figure type, and raw Fitzpatrick type

Figures that were unretrievable, duplicates, or unreadable were first excluded. Duplicates were identified by a perceptual-hash Hamming distance of 4 or less from an image already seen (106 unretrievable, 187 duplicates, 1 unreadable). Then, to automatically sort the large number of figures, automated filtering rules based on figure pixels were applied in two passes (Fig. 1). These filters were designed on properties that would not be affected by skin pigmentation. A figure was excluded as achromatic imaging, likely an MRI scan, radiograph, CT scan, or ultrasound, when at least 92% of its pixels had an RGB channel spread below 14 without the image being predominantly white, or when its pure-black pixel fraction exceeded 0.50. A figure was excluded as a chart, graph, or text-only panel when at least 75% of its pixels were near-white and chromatic content occupied less than 8% of the image, when it met the greyscale criterion while being predominantly white, or when its pure-white pixel fraction exceeded 0.40. Pure-value fractions were measured at a channel tolerance of 5 on nearest-neighbour downsampled copies so that exact pixel values were preserved. Together these rules eliminated 10,043 figures as achromatic imaging and 16,970 as charts, graphs, or text-only panels. The filtering code is provided in Supplementary File 1. Color and intensity histogram features of this kind are established discriminators of imaging modality in figures drawn from the biomedical literature, including the separation of photographs from radiographic and graphical content [16]. Furthermore, these filters would not have eliminated skin, regardless of its pigmentation, because compression noise, lighting gradients, and natural color variations ensure actual photographs of skin do not present a large fraction of pure-black or pure-white pixels.

To verify that the application of these filters was appropriate, 202 eliminated figures that fell closest to these cutoffs were identified. Of these, as deemed by the first author, only 6.9% were incorrectly flagged for exclusion and were subsequently reinstated. As these 202 figures would comprise the band closest to exclusion, and thus were the most likely to contain misclassified figures, the false-positive rate of these filtering rules across all the figures that were eliminated is likely even lower than 6.9%.

After automatic filtering, 13,367 figures remained. Since these figure counts considered each panel of a multi-panel figure separately, all figures part of a large multi-panel composite figure were collapsed to their parent published figure. In other words, if an image was made of panel A, B, and C, instead of being considered as 3 separate figures, it was counted as 1 composite figure. This step prevents pseudo replication, since multi-panel composites in orthopedics are commonly serial photographs of a single patient (i.e., preoperative, intraoperative, and postoperative views) that are not statistically independent. This yielded 4,809 figure-level units for classification.

### Subspecialty assignment

Each figure was assigned to a subspecialty based on a weighted keyword matching system applied to the article title, MeSH terms, and abstract. Nine subspecialties were defined: Shoulder & Elbow, Spine, Sports Medicine, Foot & Ankle, Hand & Wrist, Hip, Knee, Trauma/General, and Oncology/Other. Figures that did not match any subspecialty keyword criteria were labeled Unclassified. Unclassified figures were retained as a tenth category, so that ten categories are reported throughout.

### Artificial intelligence (AI) classification

All 4,809 figures were classified with GPT-4o, a flagship multimodal AI model, with the detail parameter set to high. A structured prompt returned four fields per figure in an output JSON file. This included skin presence (yes/no), figure type (clinical, intraoperative, or illustration), Fitzpatrick type (I-VI), and a confidence score (1–5). The prompt directed the model to judge constitutive pigment rather than apparent tone, and supplied explicit corrections for orthopedic confounds, including, but not limited to, betadine staining and blood. Medically documented visual anchors were provided for each Fitzpatrick type. The final prompt was modified after pilot testing revealed a central tendency bias and a tendency to read warm surgical lighting on pale skin as evidence of a higher Fitzpatrick type. Classification was performed in June 2026 through the OpenAI API using the model alias “gpt-4o”, with JSON output mode and the API default sampling parameters rather than a pinned temperature or seed. No dated model snapshot was recorded at the time of the original run. In the repeatability analysis described in Sect.  "Validation of AI classification" the same alias was served by gpt-4o-2024-08-06. Each figure was submitted once, and no figure was re-queried or averaged. Two of the 4,809 figures (0.04%) returned no usable response and were treated as containing no gradable skin. The 61 figures used for pilot prompt testing received a confidence rubric worded more verbosely than the final prompt, with identical skin presence, figure type and Fitzpatrick instructions. The verbatim prompt and all model and request parameters are provided in Supplementary File 1, together with the image filtering code.

Of the 4,809 figures, those classified as containing no gradable skin were excluded. Figures the model rated at confidence 2 or below, together with skin-positive figures for which the model returned no valid Fitzpatrick type, were manually reviewed by the first author (286 figures in total: 233 at confidence 2, 3 at confidence 1, and 50 without a valid Fitzpatrick type). Of these, 164 were reclassified as lacking gradable skin and excluded, none of which had been rated V-VI by the model, and 122 were retained. Among the 96 retained figures carrying both a model and a reviewer Fitzpatrick type, 67 were unchanged, 25 were revised darker and 4 lighter (mean + 0.31 types), and 22 changed Fitzpatrick bin, of which 3 moved into the dark bin and none moved out of it. A reviewer type was assigned to a further 25 figures for which the model returned none, and one figure type was corrected. All results reported in Sect.  "Results" reflect the dataset after this review. Without it, dark-skin representation would have been 4.35% rather than 4.84%. The final analytic dataset comprised 2,025 skin-positive figures.

### Validation of AI classification

A comparator set comprising a random sample of 481 figures (10% of all figures sent for AI analysis) was assembled. Classifier performance for Fitzpatrick skin type was validated against the independent ratings of a board-certified dermatologist. For skin presence and figure type, the model was scored against the primary author’s own labels. Agreement for Fitzpatrick type is reported both for the six-point scale and for the three collapsed bins on which every analysis in this paper is based, with Cohen’s kappa for the binned comparison and quadratic-weighted kappa for the six-point scale. Repeatability was assessed separately by re-classifying a random sample of 200 analytic figures under the same model alias, prompt, detail setting and sampling parameters as the original run.

### Analysis

Results were analyzed using three Fitzpatrick bins, with Type I and II representing light skin tones, Type III and IV representing medium skin tones, and Type V and VI representing dark skin tones, which are classifications supported by recent literature [17]. Grouping adjacent types reduces the impact of exact-match inaccuracies stemming from the classification pipeline while preserving clinically meaningful skin tone distinctions. Illustrations were excluded from figure type comparisons because too few survived screening to support adequately powered analysis (*n* = 26). The small number of surviving illustrations is an expected consequence of the pure-white pixel filter, which removes line drawings rendered on white backgrounds, so the filtering design trades illustration coverage for reliable exclusion of charts and text panels. Country of origin was assigned to each article based on author affiliation data, defaulting to the corresponding author’s affiliation and falling back to the first author’s affiliation. Although this is not always perfect, it is a well-established method that has been applied in similar studies [18, 19]. If affiliation data was not interpretable, the figures from that article were excluded from geographic analysis. Because collapsing multiple figures from the same article into a single per-article median did not significantly change the results, subsequent analyses were performed at the per-figure level. Because 74 hypothesis tests are reported in this manuscript, p values were adjusted using the Benjamini-Hochberg false discovery rate procedure. Unadjusted p values are reported throughout and comparisons that did not remain significant after adjustment are identified as such (Supplementary Table 2).

The observed distribution was compared against two population references. The diverse-population reference (Light 16%, Medium 40%, Dark 44%) is the whole-sample Fitzpatrick phototype distribution reported by He et al. in 3,386 adults recruited to be ethnically diverse (70% non-White) [20]. Because no population-representative global Fitzpatrick survey exists, and because reference distributions used in imaging research are frequently undocumented [21], this distribution was used as a proxy for a globally diverse population and its derivation is reported in full. Weighting the race-specific distributions of He et al. by world regional population (United Nations World Population Prospects 2024) yields Light 11%, Medium 37%, Dark 52%, indicating that the reference, if anything, understates the global share of darker phototypes. The United States reference (Light 34.7%, Medium 52.7%, Dark 12.6%) is the survey-weighted distribution of dermatologist-graded digital image skin types in 2,691 adults aged 20–59 from NHANES 2003–2004 [22]. Because skin type in that survey was graded from standardized photographs rather than self-reported [23], it measures the same construct as the present study. The derivation of both references, together with a US Census-weighted sensitivity estimate, is given in Supplementary Table 3.

## Results

### Validation of the AI classification pipeline

AI skin detection had a sensitivity of 99.3% and specificity of 79.4% (PPV 65.5%, NPV 99.6%; *n* = 481), and figure-type classification was 89.0% accurate overall (94.4% clinical, 86.1% intraoperative; *n* = 136). For Fitzpatrick typing, the AI and the dermatologist agreed exactly in 45.7% of cases and agreed within one type in 90.7% of cases (*n* = 129), with a tendency for the model to rate skin 0.39 types darker than the dermatologist. For the three bins used in all subsequent analyses, exact agreement with the dermatologist was 67.4% (Cohen’s κ = 0.22, 95% CI 0.06–0.37, and linear-weighted κ = 0.28), with a quadratic-weighted κ of 0.47 (0.28–0.62) for the six-point scale. Kappa is depressed here by highly unbalanced marginals, as 81% of the validation figures were light-skinned, and disagreement was directional rather than random: the model classified 4.7% of the validation set as dark compared with 3.1% by the dermatologist. On re-classification of 200 randomly selected analytic figures under identical conditions, agreement with the original run was 99.0% for skin presence (95% CI 96.4–99.7), 94.9% for figure type (91.0-97.2) and 87.2% for the three bins (81.8–91.2, κ = 0.74), with 69.4% exact and 97.4% within-one-type agreement on the six-point scale. No figure moved between the light and dark bins, changes were symmetric (33 darker and 27 lighter, mean shift + 0.05 types), and the estimated dark-skin proportion was 6.0% in the original run versus 5.0% on repeat (McNemar *p* = 0.63).

### Overall skin tone distribution

**Fig. 2:**
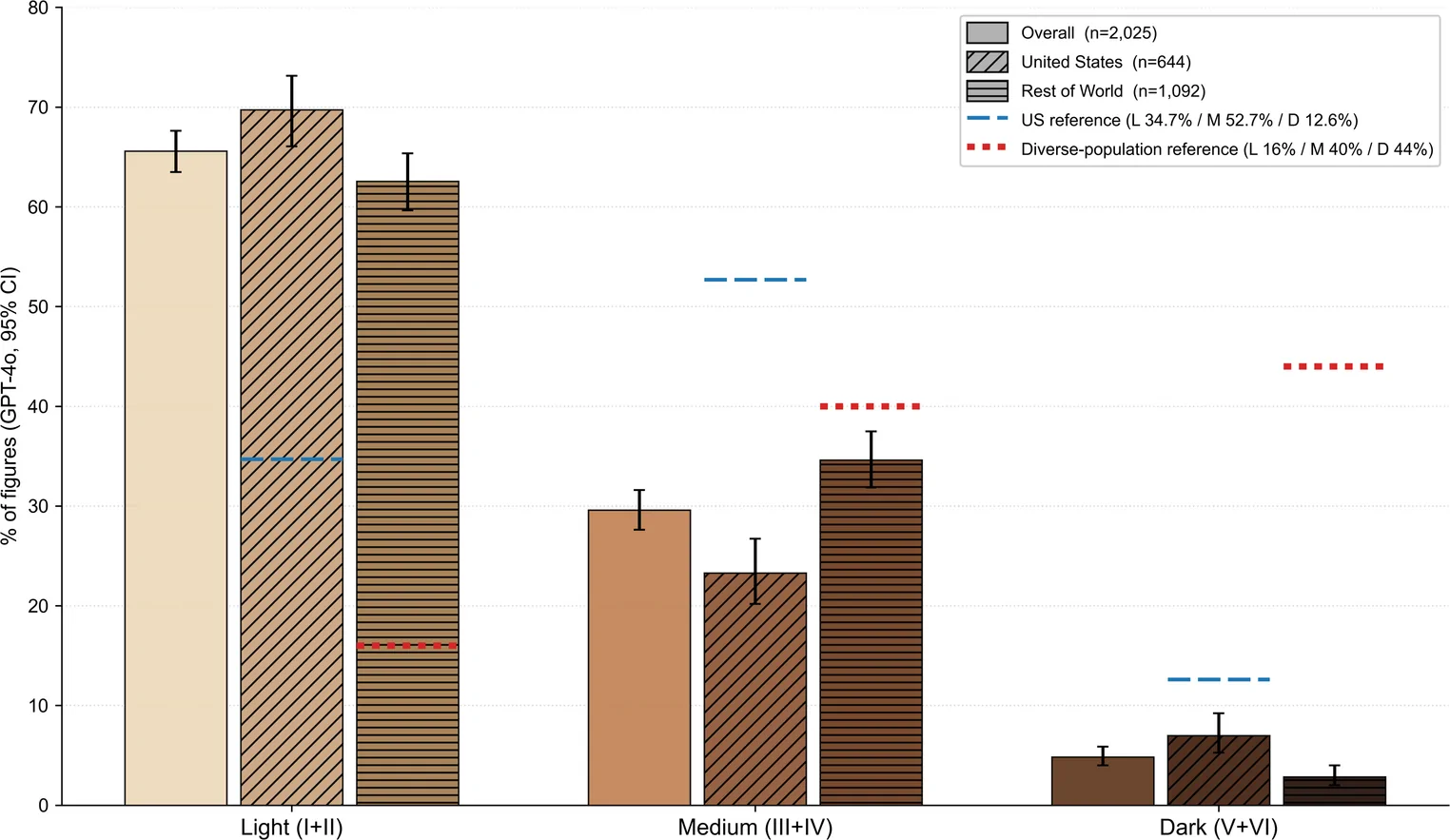
Light, medium, and dark skin tone representation overall and by the United States versus the Rest of World (RoW). Bars show the percentage of figures with 95% CIs, and dashed lines mark the US (blue) and diverse-population (red) reference values

Across all included figures, light skin (Fitzpatrick I-II) accounted for 65.58% of images (95% CI 63.48–67.62%), medium skin (III-IV) for 29.58% (27.63–31.61%), and dark skin (V-VI) for 4.84% (3.99–5.86%) (Fig. 2). Relative to both the diverse-population reference and the US reference, light skin was significantly overrepresented (*p* < 0.001), while medium skin and dark skin were significantly underrepresented (*p* < 0.001). Because published population references vary, we also note that the upper exact 95% confidence limit for dark-skin representation was 5.9%, so the deficit would remain significant against any reference exceeding 5.9% Fitzpatrick V-VI.

### Representation across subspecialties and figure type

**Fig. 3:**
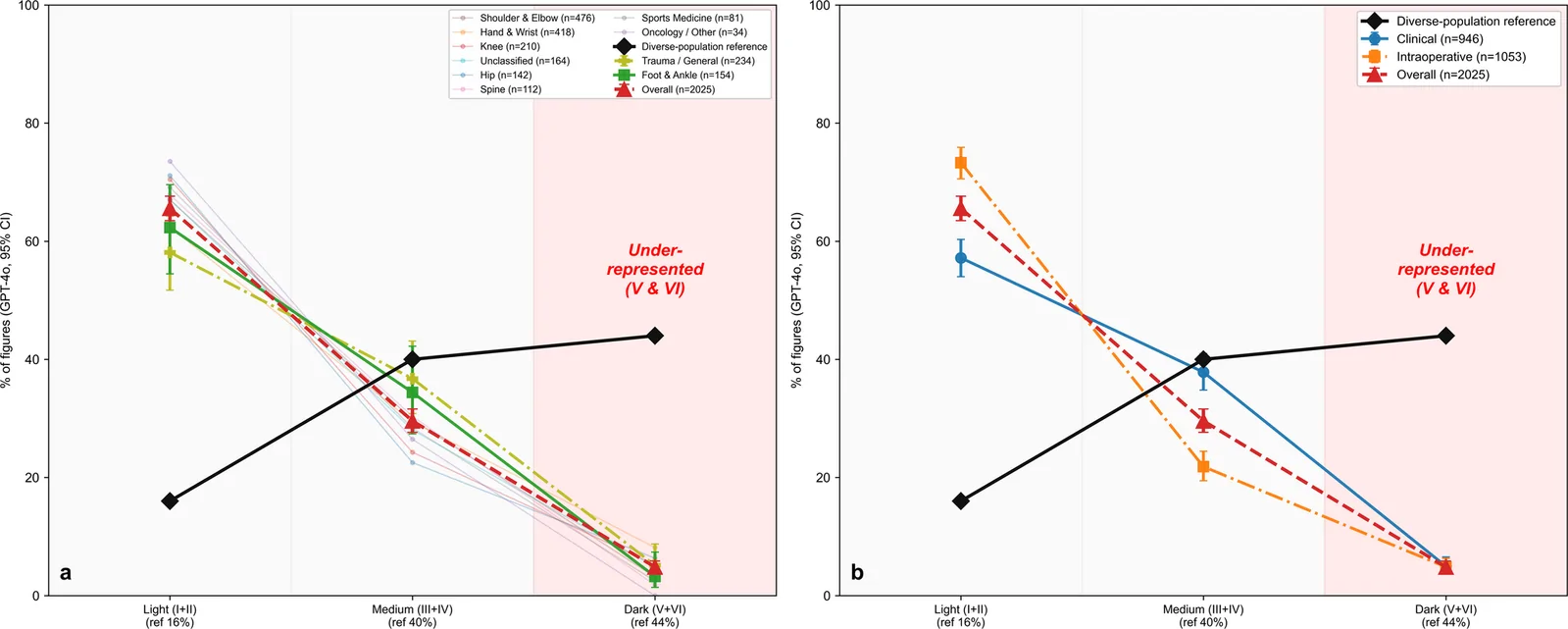
Skin tone distribution across Fitzpatrick bins, plotted against the diverse-population reference distribution (black diamonds), shown by **a** orthopedic subspecialty and **b** figure type (clinical versus intraoperative)

Underrepresentation of dark skin was universal across all nine subspecialties and the Unclassified group, with each falling significantly below the diverse-population reference (each *p* < 0.001), ranging from 0% (Oncology/Other) to 8.1% (Hand & Wrist), and no subspecialty’s confidence interval approached the 44% reference (Fig. 3a). Similarly, light skin was significantly overrepresented in every subspecialty (each *p* < 0.001). The medium skin deficit was not universal. Trauma/General (36.8%, *p* = 0.31) and Foot & Ankle (34.4%, *p* = 0.16), did not significantly differ from the diverse-population medium reference. Spine (30.4%, *p* = 0.037) did not remain significant after correction for multiple comparisons (q = 0.054).

Clinical and intraoperative photographs were statistically indistinguishable in dark skin representation (5.0% vs. 4.8%; *p* = 0.98). Clinical photographs contained significantly more medium skin (37.8% vs. 21.8%; χ² = 60.7, *p* < 0.001) and significantly less light skin (57.2% vs. 73.3%; χ² = 56.8, *p* < 0.001) than intraoperative photographs (Fig. 3b).

### Geographic patterns

US-authored and rest-of-world (RoW) figures differed significantly in all three bins (Fig. 2). RoW figures contained more medium skin (34.6% vs. 23.3%, *p* < 0.001), whereas US figures contained more dark skin (7.0% vs. 2.8%, *p* < 0.001) and more light skin (69.7% vs. 62.5%, *p* = 0.003). Both groups still significantly overrepresented light skin tones (US vs. US reference: χ² = 157.5, *p* < 0.001; RoW vs. diverse-population reference: χ² = 491.7, *p* < 0.001) and underrepresented medium (US: χ² = 116.5, *p* < 0.001; RoW: χ² = 6.6, *p* = 0.010) and dark skin tones (US: χ² = 10.8, *p* = 0.001; RoW: χ² = 512.7, *p* < 0.001). These comparisons retained the same direction and significance against the world-weighted and US Census-weighted sensitivity references (Supplementary Table 3), with the single exception of RoW medium skin against the world-weighted reference (34.6% vs. 37.4%, *p* = 0.061).

Out of the high-volume non-US literatures, several met the diverse-population medium reference (China 41.1%, *p* = 0.73; South Korea 40.0%, *p* = 1.0; Brazil 47.6%, *p* = 0.48; India 63.3%, *p* = 0.001 in the overrepresented direction), but none met the diverse-population dark reference.

### Temporal trend

**Fig. 4:**
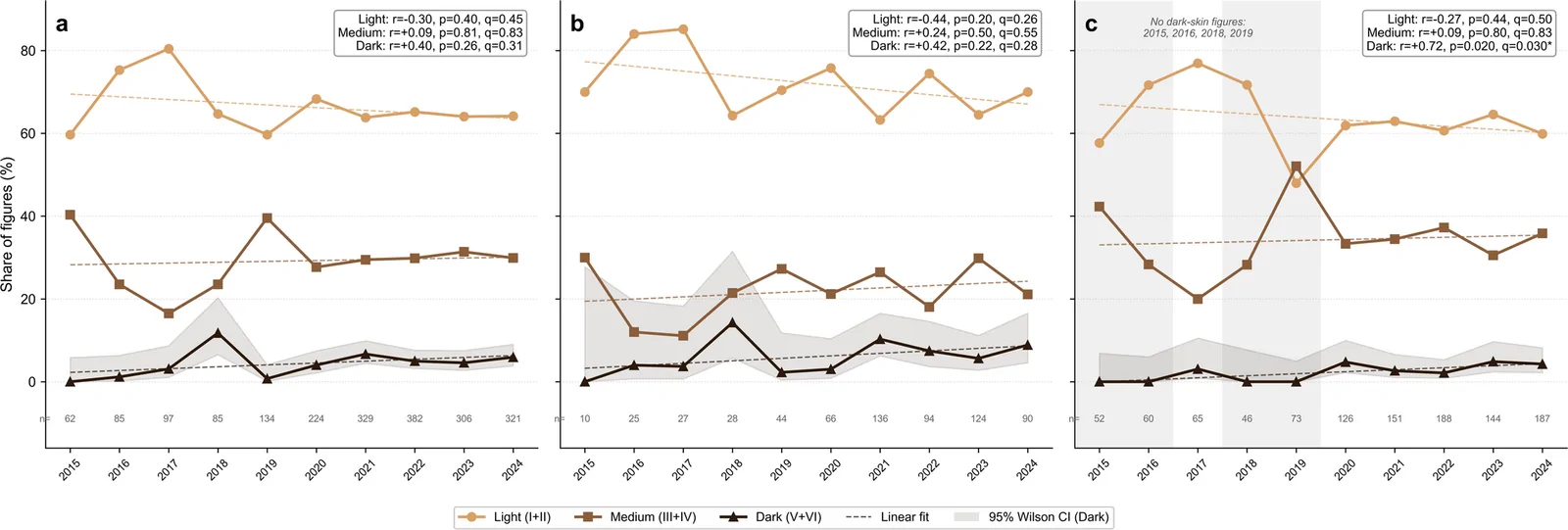
Yearly share of figures in each Fitzpatrick bin from 2015 to 2024 for (**a**) the overall sample, (**b**) United States affiliated figures, and (**c**) RoW figures. The shaded band is the 95% Wilson confidence interval for the dark skin trend, and per-year sample sizes are shown along the x-axis. The statistically significant finding (panel c) is marked by an asterisk (*), though the low dark-skin count in early years may be artificially inflating the statistic

Temporal trend analysis across the 10-year study period (2015–2024) revealed no significant change in overall dark skin representation (logistic OR = 1.092/year, *p* = 0.057) (Fig. 4). Light and medium skin tone proportions were similarly stable across all trend tests (all *p* > 0.39). In geographic subgroup analyses, US authored figures showed no significant temporal trend for any skin tone category (dark skin: OR = 1.073/year, *p* = 0.329). However, RoW figures demonstrated a significant increase in dark skin representation over time (*r* = 0.716, *p* = 0.020; OR = 1.224/year, *p* = 0.019), corresponding to an approximately 22% relative increase in the odds of a dark skin figure per calendar year. The year-by-region interaction term was not significant for any skin tone bin, however (dark: OR = 1.14 per year, 95% CI 0.91–1.42, *p* = 0.24), indicating that the apparent increase in RoW dark-skin representation cannot be distinguished statistically from the non-significant US trend. The US subgroup comprised 644 figures (45 dark) against 1,092 RoW figures (31 dark), with as few as 10 to 28 US figures per year between 2015 and 2018, limiting power to detect a US-specific trend. This trend should be interpreted cautiously, however, as it was driven in part by sparse early year counts, including four of the first five study years (2015, 2016, 2018 and 2019) with 0% dark skin representation in RoW figures.

## Discussion

To our knowledge, this is the first study to evaluate such a large volume of orthopedic figures, assess the representation gap within open-access journals specifically, and determine whether this gap persists across orthopedic subspecialties, figure type, and into more recent years. By addressing these previously unanswered dimensions of skin of color representation in orthopedic literature, this study seeks to deepen understanding of the extent and impact of the representation-gap.

Across open-access orthopedic literature, skin tone representation significantly overrepresents lighter skin tones and significantly underrepresents darker skin tones compared to both US and diverse-population references. Light skin (Fitzpatrick I-II) accounted for over 65% of the 2,025 analyzed figures, while dark skin (Fitzpatrick V-VI) made up less than 5%. Medium skin tones were also underrepresented overall, though less uniformly across subspecialties. These findings align with Ferrell et al., who examined 649 figures in four paywalled orthopedic journals and similarly found significant overrepresentation of Fitzpatrick types I-III and significant underrepresentation of Fitzpatrick types IV-VI [15]. However, while the identified dark-skin underrepresentation in our study was of comparable magnitude to that reported by Ferrell et al., we found light-skin overrepresentation was more pronounced in our data (65.6% observed vs. 35.0% expected, compared with their 87.0% observed vs. 61.7% expected). The fact that the representation-gap is steeper in open-access journals than in paywalled journals is especially concerning because open access content potentially reaches a broader audience, therefore amplifying the educational impact of this less representative imagery. In fact, Davis et al. found that open-access articles received 89% more full-text downloads and 23% more unique visitors than subscription articles, with Yi et al. finding open-access articles in Postgraduate Medical Journal had significantly higher page views, citations, and downloads compared to subscription articles [24, 25]. Additionally, Vadhera et al. found that in orthopedics specifically, open access articles have a mean citation rate nearly double that of subscription articles and receive significantly greater social media attention [26]. Representative imagery published in open access journals is not only freely available, but more widely disseminated. Clinically, this propagation of deeply misrepresentative skin of color figures to a wide audience may not only impede clinical training, but also shape implicit and incorrect assumptions about what a “typical” orthopedic patient looks like [5].

On a subspecialty breakdown, all nine subspecialties, as well as the Unclassified group, significantly underrepresented dark skin types. Only three subspecialties, trauma/general, foot & ankle and spine, did not significantly underrepresent medium skin types after correction for multiple comparisons. This may be explained by the fact that orthopedic trauma populations are demographically broad, and minoritized patients carry a disproportionate share of lower extremity injury and polytrauma [27]. Foot and ankle care similarly draws heavily on diabetic limb-salvage populations, in which Black and Hispanic patients are overrepresented [28]. Another explanation is that both lean heavily on non-elective care, and minoritized patients are disproportionately represented in this kind of care, since they are less likely to undergo elective procedures and are more likely to present emergently, often later in the disease course [29]. However, these subspecialties are exceptions, and not the norm. No subspecialty was broadly representative, and adequate medium-skin representation never extended to the dark end of the spectrum. Thus, orthopedics faces a systemic representational gap that spans the entire field, such that no single subspecialty training pathway can compensate for it through routine literature exposure alone.

Regarding figure type, clinical photographs contained more medium skin and less light skin than intraoperative images. This comparatively more balanced representation across the light and medium range is a promising finding, since clinical photographs have been the dominant format for teaching clinical pattern recognition since the mid-20th century [30, 31]. However, clinical and intraoperative photographs still both significantly underrepresented dark skin, which is where visual pattern recognition is most unreliable and image-based training is most needed [10, 32].

The geographic pattern showed that the deficit is global. Both US authored figures and RoW figures significantly overrepresented light skin tones while significantly underrepresenting medium and dark skin tones. Interestingly, the US had relatively more dark skin tone representation than the RoW. This may reflect the increasingly diverse US population, which is also less racially homogenous compared to other major publishing countries [1, 33]. Additionally, there have been US specific institutional policies that have fostered greater attention to racial representation, such as the American Orthopaedic Association’s designation of workforce diversity as a critical issue, which do not have direct equivalents internationally [34]. On a per-country basis, out of those with an adequate sample size, only India had a distribution that approximated the combined medium-and-dark share of the diverse-population reference (77.6% observed vs. 84% expected), which is consistent with their predominantly medium and darker toned populations. Still, since the shortfall in dark skin representation generally persists across national literatures, broadening the geographic scope of one’s reading would not yield more representative visual training material, and thus is a problem that cannot be addressed at the level of readership.

Finally, the temporal analysis showed no measurable progress overall, which aligns with Ferrell et al., who similarly found no significant change in darker skin tone representation in paywalled orthopedic journals in 2010 versus in 2020. The fact that this trend was observed between two independent datasets, different journal types, and different time windows makes it clear that the representational deficit is not improving over time. However, geographic subgroup findings diverge in an instructive way. Ferrell et al. found a significant increase in darker skin representation over time in US-based articles and no change internationally, whereas we found a significant temporal increase in international figures over time but not in US figures. The year-by-region interaction in our data was not significant (*p* = 0.24), however, so our subgroup results are presented as exploratory and do not establish a difference between regions. The contrast with Ferrell et al. reflects what each design was powered to detect rather than an established reversal. One contributing factor may be the greater methodological granularity of the present analysis. Ferrell et al. compared only two snapshot years (2010 and 2020), which is susceptible to year-specific sampling variation, whereas the present study modeled continuous trends across 10 annual data points, providing a more robust assessment of temporal trajectory. It is worth noting, however, that the international trend identified here, while statistically significant, arose from a low baseline of four of the first five study years of 0% dark skin representation in RoW figures, so even modest absolute increases produced large relative effects. Still, the clinical reality remains unchanged that in both data sets, dark skin representation in the most recent years remains far below population benchmarks, and the current rate of change is insufficient to close the gap within any clinically meaningful timeframe. A decade of growing awareness, diversity initiatives, and editorial statements has not translated into measurable change in published imagery [2]. Therefore, embedding representation into editorial policy, rather than leaving it to individual authors, may be the more plausible route to durable change. Journals could adopt explicit representation benchmarks for submitted clinical images, encourage authors to embrace standardized skin tone reporting requirements, and audit published figures periodically using automated tools of the kind applied here [4, 35].

There are several limitations to this study. First, the Fitzpatrick scale was originally developed to measure UV erythema response rather than constitutive pigmentation. It is thus an imperfect measurement for skin tone. Nonetheless, it has since become the most widely used skin classification system in dermatologic and medical education research and is frequently employed as a proxy for skin tone [36, 37]. Additionally, it was selected to maintain consistency with prior orthopedic skin tone analyses and it remains the standard used in contemporary skin representation studies [17]. However, future studies could adopt the more granular and inclusive Monk Skin Tone scale, which was introduced in more recent years [38]. Second, despite being validated, the AI classifier naturally has limits. The model rated skin about 0.39 types darker than the dermatologist on average, and although agreement within one type was high at 90.7%, exact agreement was modest at 45.7%, so type-level precision is constrained. Chance-corrected agreement for the three analysis bins was also low (κ = 0.22), reflecting both the highly unbalanced marginals of the validation set, in which 81% of figures were light-skinned, and the fact that disagreement was systematic rather than random. However, the discrepancy matters little for our conclusions, both because all figures were collapsed into two-type bins and no exact-type analysis was performed, and because the central finding is robust to exact-type inaccuracies. Even under a uniform one-type darkening shift, moving all type IV to V and all type V to VI, dark skin would remain significantly underrepresented. If anything, the model’s 0.39 darker bias means our estimates of dark skin representation are already conservative overestimates, and the deficit might even be more pronounced. Third, the reference distributions and the present figures do not measure skin tone in the same way. He et al. recorded self-reported sun-reactive phototype, whereas this study graded apparent constitutive pigmentation from photographs, and self-report tends to shift toward darker types, with 17.8% of White respondents reporting types V-VI [23]. The reference may therefore overstate dark-type prevalence relative to what photographic grading would find. Weighting He et al. by world population points the other way, suggesting the reference understates the global dark share. Because these considerations act in opposite directions, we also report a reference-free threshold: the upper 95% confidence limit for dark-skin representation was 5.9% overall, 9.2% in US figures and 4.0% in RoW figures, so the deficit holds against any population reference exceeding those values. Fourth, figures were included whenever gradable skin was visible, and we did not classify whether interpreting a given figure actually requires the reader to judge the skin. Much orthopedic imagery in which skin is visible depicts angular or rotational deformity, swelling, limb position after injury, arthritic deformity, contracture, range of motion, gait and alignment, surgical approaches, or healed incisions, where the interpretive target is bone, joint, alignment, or operative technique rather than pigmentation. Intraoperative photographs, which make up a substantial share of the present sample, are the clearest case, since the field is prepped and draped. Our results therefore establish that the published visual record of orthopedics is skewed toward light skin, and that this skew shapes the implicit norm of who an orthopedic patient appears to be. They do not by themselves quantify representation within the narrower set of figures in which recognition depends directly on skin tone, such as erythema and its borders, cellulitis, surgical site or deep infection, wound healing and dehiscence, ischemia, mottling, and blistering. Measuring representation inside that pigment-dependent subset is the natural next step, and the pipeline described here can be extended with a field capturing whether figure interpretation requires assessment of the skin itself. Finally, GPT-4o is a proprietary model that does not guarantee identical output on repeated classification and may not remain available in its present form, and model and dataset details in imaging research are frequently underreported [21]. Classification was run with the API default sampling parameters rather than a pinned temperature or seed. Repeat classification of 200 figures nonetheless reproduced the original three-bin label in 87.2% of cases (κ = 0.74) and the estimated dark-skin proportion to within 1% point (6.0% versus 5.0%, McNemar *p* = 0.63), so run-to-run variation is small relative to the gap between roughly 5% and any population reference. The verbatim prompt, model identifiers, image filtering code and the per-figure results of the repeatability analysis are provided in Supplementary Files , so that the pipeline can be reproduced with this or a successor model.

## Conclusion

Using a large-scale computational pipeline validated against a board-certified dermatologist, we found that orthopedic imagery markedly overrepresents light skin and underrepresents medium and dark skin relative to both a diverse-population reference and US population estimates in open-access literature. The dark skin underrepresentation holds across every orthopedic subspecialty, every figure type, and the full decade studied, with no evidence of meaningful improvement over time. Together, these results show that the published visual record of orthopedic surgery does not reflect the patients the specialty treats, which narrows the implicit standard of what an orthopedic patient looks like and limits the material available for teaching findings whose appearance depends on skin tone. Closing the representation-gap will require deliberate editorial action rather than continued reliance on awareness alone.

## Supplementary Information

Below is the link to the electronic supplementary material.

Supplementary Material 1

Supplementary Material 2

Supplementary Material 3

Supplementary Material 4

Supplementary Material 5

## Data Availability

The figures analyzed in this study are drawn from publicly available, open-access articles in the peer-reviewed literature and can be accessed directly through the original publications.
