# Supplementary material for: Skin color representation in orthopedic literature
Source: Arch Orthop Trauma Surg. 2026 Nov 3;146(1):392. doi: 10.1007/s00402-026-06561-4 (PMC13731530; doi:10.1007/s00402-026-06561-4)
Supplement: Supplementary file 1 [file 402_2026_6561_MOESM1_ESM.docx]

*Skin Color Representation in Orthopedic Literature. Archives of Orthopaedic and Trauma Surgery. Electronic supplementary material.*

**Supplementary Table 1. Journals sampled and search strategy**

| **Journal (NLM Catalog title) [NLM abbreviation; ISSN]** | **Years with articles** | **Articles found** | **Articles sampled** | **Journal-year cells capped** | **Articles with figures** | **Figures harvested** | **Figures to AI** | **Figures analysed** |
| --- | --- | --- | --- | --- | --- | --- | --- | --- |
| Journal of Orthopaedic Surgery and Research [J Orthop Surg Res; ISSN 1749-799X (e)] | 2015-2024 | 4,613 | 750 | 10 | 717 | 7,965 | 742 | 228 |
| BMC Musculoskeletal Disorders [BMC Musculoskelet Disord; ISSN 1471-2474 (e)] | 2015-2024 | 7,138 | 750 | 10 | 690 | 5,496 | 525 | 165 |
| Arthroplasty Today [Arthroplast Today; ISSN 2352-3441 (e)] | 2015-2024 | 1,379 | 671 | 7 | 531 | 3,698 | 568 | 178 |
| JB & JS Open Access [JB JS Open Access; ISSN 2472-7245 (e)] | 2016-2024 | 476 | 449 | 3 | 366 | 3,055 | 242 | 88 |
| Journal of the American Academy of Orthopaedic Surgeons. Global Research & Reviews [J Am Acad Orthop Surg Glob Res Rev; ISSN 2474-7661 (e)] | 2017-2024 | 860 | 535 | 6 | 418 | 2,806 | 286 | 135 |
| Orthopaedic Journal of Sports Medicine [Orthop J Sports Med; ISSN 2325-9671 (e)] | 2015-2024 | 5,424 | 750 | 10 | 461 | 2,782 | 296 | 86 |
| JSES International [JSES Int; ISSN 2666-6383 (e)] | 2019-2024 | 989 | 390 | 5 | 348 | 2,598 | 335 | 135 |
| Journal of Hand Surgery Global Online [J Hand Surg Glob Online; ISSN 2589-5141 (e)] | 2019-2024 | 563 | 357 | 3 | 299 | 2,525 | 574 | 426 |
| JSES Reviews, Reports, and Techniques [JSES Rev Rep Tech; ISSN 2666-6391 (e)] | 2020-2024 | 417 | 312 | 4 | 294 | 2,438 | 623 | 369 |
| Bone & Joint Open [Bone Jt Open; ISSN 2633-1462 (e)] | 2020-2024 | 619 | 375 | 5 | 305 | 2,375 | 141 | 38 |
| North American Spine Society Journal [N Am Spine Soc J; ISSN 2666-5484 (e)] | 2020-2024 | 335 | 308 | 3 | 254 | 2,097 | 163 | 39 |
| Clinical Orthopaedics and Related Research (open-access subset) [Clin Orthop Relat Res; ISSN 0009-921X (p); 1528-1132 (e)] | 2015-2024 | 164 | 164 | 0 | 137 | 1,236 | 83 | 17 |
| Foot & Ankle Orthopaedics [Foot Ankle Orthop; ISSN 2473-0114 (e)] | 2019-2024 | 2,342 | 442 | 5 | 253 | 1,140 | 162 | 77 |
| Hand: Official Journal of the American Association for Hand Surgery [Hand (N Y); ISSN 1558-9447 (p); 1558-9455 (e)] | 2015-2024 | 86 | 86 | 0 | 71 | 463 | 69 | 44 |
| **Total** | **2015-2024** | **25,405** | **6,339** | **71** | **5,144** | **40,674** | **4,809** | **2,025** |

**PubMed Central query template (one query per journal-year cell, NCBI E-utilities esearch, db=pmc):**

"<journal>"[Journal] AND <year>[pdat] AND "open access"[filter] AND ((("Case Reports"[pt] OR "Review"[pt] OR "Clinical Study"[pt] OR "Comparative Study"[pt] OR "Guideline"[pt] OR "Practice Guideline"[pt]) OR ("technical note"[tiab] OR "surgical technique"[tiab] OR "operative technique"[tiab] OR technique[ti] OR "how i do it"[tiab] OR "case report"[tiab] OR "case series"[tiab] OR "outcomes"[tiab])))

**Article-type string (PUBTYPE_TERMS), in full:**

(("Case Reports"[pt] OR "Review"[pt] OR "Clinical Study"[pt] OR "Comparative Study"[pt] OR "Guideline"[pt] OR "Practice Guideline"[pt]) OR ("technical note"[tiab] OR "surgical technique"[tiab] OR "operative technique"[tiab] OR technique[ti] OR "how i do it"[tiab] OR "case report"[tiab] OR "case series"[tiab] OR "outcomes"[tiab]))

Sampling: 140 journal-year cells (14 journals x 2015-2024). For each cell, the random generator was seeded with the string "20250601|<journal>|<year>" (Python random.Random), so each cell’s draw is reproducible independently of the others. Cell cap: 75 articles.

**Footnotes**

(a) Articles sampled (6,339) exceeds the 5,144 articles cited in the main text because 1,195 sampled articles contained no downloadable figures.

(b) Journals launched after 2015 contribute fewer years; years with articles are shown per journal.

(c) Where a journal-year cell returned more than 75 eligible articles, 75 were drawn by simple random sampling without replacement using the fixed seed above; otherwise all eligible articles were included.

*Figures to AI = after automated image filtering and panel collapse. Figures analysed = skin-positive with a valid Fitzpatrick type after manual review.*
