# Supplementary material for: Skin color representation in orthopedic literature
Source: Arch Orthop Trauma Surg. 2026 Nov 3;146(1):392. doi: 10.1007/s00402-026-06561-4 (PMC13731530; doi:10.1007/s00402-026-06561-4)
Supplement: Supplementary file 2 [file 402_2026_6561_MOESM2_ESM.docx]

*Skin Color Representation in Orthopedic Literature. Archives of Orthopaedic and Trauma Surgery. Electronic supplementary material.*

**Supplementary Table 2. Multiple-comparison correction**

Benjamini-Hochberg false-discovery-rate correction was applied across all 74 hypothesis tests reported in the manuscript, treated as a single family: overall (6), subspecialty (30), figure type (3), regional (9), per-country (8), temporal (18). 51 tests were significant before correction (p < 0.05) and 50 after (q < 0.05). The single change in status, Spine medium skin vs the diverse-population reference, is highlighted.

*References: diverse-population reference = He et al. 2014, Light 16% / Medium 40% / Dark 44%; US reference = Keiser et al. 2012 (NHANES 2003-2004), Light 34.7% / Medium 52.7% / Dark 12.6%. Rows are grouped by family and sorted by raw p within family.*

| **Family** | **Comparison** | **Test** | **Statistic** | **Raw p** | **BH q** | **Significant at q < 0.05** |
| --- | --- | --- | --- | --- | --- | --- |
| Overall | All figures, light skin vs diverse-population reference | Chi-square goodness of fit | chi2(1) = 3703.8 | <0.001 | <0.001 | Yes |
| Overall | All figures, dark skin vs diverse-population reference | Chi-square goodness of fit | chi2(1) = 1260.3 | <0.001 | <0.001 | Yes |
| Overall | All figures, light skin vs US reference | Chi-square goodness of fit | chi2(1) = 852.2 | <0.001 | <0.001 | Yes |
| Overall | All figures, medium skin vs US reference | Chi-square goodness of fit | chi2(1) = 434.2 | <0.001 | <0.001 | Yes |
| Overall | All figures, dark skin vs US reference | Chi-square goodness of fit | chi2(1) = 110.7 | <0.001 | <0.001 | Yes |
| Overall | All figures, medium skin vs diverse-population reference | Chi-square goodness of fit | chi2(1) = 91.6 | <0.001 | <0.001 | Yes |
| Subspecialty | Shoulder & Elbow, light skin vs diverse-population reference | Chi-square goodness of fit | chi2(1) = 921.8 | <0.001 | <0.001 | Yes |
| Subspecialty | Hand & Wrist, light skin vs diverse-population reference | Chi-square goodness of fit | chi2(1) = 670.8 | <0.001 | <0.001 | Yes |
| Subspecialty | Knee, light skin vs diverse-population reference | Chi-square goodness of fit | chi2(1) = 463.7 | <0.001 | <0.001 | Yes |
| Subspecialty | Shoulder & Elbow, dark skin vs diverse-population reference | Chi-square goodness of fit | chi2(1) = 322.3 | <0.001 | <0.001 | Yes |
| Subspecialty | Hip, light skin vs diverse-population reference | Chi-square goodness of fit | chi2(1) = 321.1 | <0.001 | <0.001 | Yes |
| Subspecialty | Unclassified, light skin vs diverse-population reference | Chi-square goodness of fit | chi2(1) = 318.3 | <0.001 | <0.001 | Yes |
| Subspecialty | Trauma / General, light skin vs diverse-population reference | Chi-square goodness of fit | chi2(1) = 308.9 | <0.001 | <0.001 | Yes |
| Subspecialty | Foot & Ankle, light skin vs diverse-population reference | Chi-square goodness of fit | chi2(1) = 246.0 | <0.001 | <0.001 | Yes |
| Subspecialty | Spine, light skin vs diverse-population reference | Chi-square goodness of fit | chi2(1) = 224.1 | <0.001 | <0.001 | Yes |
| Subspecialty | Hand & Wrist, dark skin vs diverse-population reference | Chi-square goodness of fit | chi2(1) = 218.2 | <0.001 | <0.001 | Yes |
| Subspecialty | Sports Medicine, light skin vs diverse-population reference | Chi-square goodness of fit | chi2(1) = 170.2 | <0.001 | <0.001 | Yes |
| Subspecialty | Trauma / General, dark skin vs diverse-population reference | Chi-square goodness of fit | chi2(1) = 143.5 | <0.001 | <0.001 | Yes |
| Subspecialty | Knee, dark skin vs diverse-population reference | Chi-square goodness of fit | chi2(1) = 128.1 | <0.001 | <0.001 | Yes |
| Subspecialty | Foot & Ankle, dark skin vs diverse-population reference | Chi-square goodness of fit | chi2(1) = 103.8 | <0.001 | <0.001 | Yes |
| Subspecialty | Unclassified, dark skin vs diverse-population reference | Chi-square goodness of fit | chi2(1) = 101.9 | <0.001 | <0.001 | Yes |
| Subspecialty | Oncology / Other, light skin vs diverse-population reference | Chi-square goodness of fit | chi2(1) = 83.7 | <0.001 | <0.001 | Yes |
| Subspecialty | Hip, dark skin vs diverse-population reference | Chi-square goodness of fit | chi2(1) = 81.7 | <0.001 | <0.001 | Yes |
| Subspecialty | Spine, dark skin vs diverse-population reference | Chi-square goodness of fit | chi2(1) = 81.0 | <0.001 | <0.001 | Yes |
| Subspecialty | Sports Medicine, dark skin vs diverse-population reference | Chi-square goodness of fit | chi2(1) = 56.7 | <0.001 | <0.001 | Yes |
| Subspecialty | Oncology / Other, dark skin vs diverse-population reference | Chi-square goodness of fit | chi2(1) = 26.7 | <0.001 | <0.001 | Yes |
| Subspecialty | Knee, medium skin vs diverse-population reference | Chi-square goodness of fit | chi2(1) = 21.6 | <0.001 | <0.001 | Yes |
| Subspecialty | Shoulder & Elbow, medium skin vs diverse-population reference | Chi-square goodness of fit | chi2(1) = 20.5 | <0.001 | <0.001 | Yes |
| Subspecialty | Hand & Wrist, medium skin vs diverse-population reference | Chi-square goodness of fit | chi2(1) = 19.5 | <0.001 | <0.001 | Yes |
| Subspecialty | Hip, medium skin vs diverse-population reference | Chi-square goodness of fit | chi2(1) = 18.0 | <0.001 | <0.001 | Yes |
| Subspecialty | Unclassified, medium skin vs diverse-population reference | Chi-square goodness of fit | chi2(1) = 9.8 | 0.002 | 0.003 | Yes |
| Subspecialty | Sports Medicine, medium skin vs diverse-population reference | Chi-square goodness of fit | chi2(1) = 4.5 | 0.033 | 0.049 | Yes |
| Subspecialty | Spine, medium skin vs diverse-population reference | Chi-square goodness of fit | chi2(1) = 4.3 | 0.037 | 0.054 | No (changed) |
| Subspecialty | Oncology / Other, medium skin vs diverse-population reference | Chi-square goodness of fit | chi2(1) = 2.6 | 0.107 | 0.146 | No |
| Subspecialty | Foot & Ankle, medium skin vs diverse-population reference | Chi-square goodness of fit | chi2(1) = 2.0 | 0.157 | 0.208 | No |
| Subspecialty | Trauma / General, medium skin vs diverse-population reference | Chi-square goodness of fit | chi2(1) = 1.0 | 0.311 | 0.371 | No |
| Figure type | Clinical vs intraoperative, medium skin | Chi-square 2x2 (Yates) | chi2(1) = 60.7 | <0.001 | <0.001 | Yes |
| Figure type | Clinical vs intraoperative, light skin | Chi-square 2x2 (Yates) | chi2(1) = 56.8 | <0.001 | <0.001 | Yes |
| Figure type | Clinical vs intraoperative, dark skin | Chi-square 2x2 (Yates) | chi2(1) = 0.0 | 0.980 | 0.993 | No |
| Regional | Rest of World figures, dark skin vs diverse-population reference | Chi-square 2x2 vs reference (Yates) | chi2(1) = 512.7 | <0.001 | <0.001 | Yes |
| Regional | Rest of World figures, light skin vs diverse-population reference | Chi-square 2x2 vs reference (Yates) | chi2(1) = 493.4 | <0.001 | <0.001 | Yes |
| Regional | US figures, light skin vs US reference | Chi-square 2x2 vs reference (Yates) | chi2(1) = 157.5 | <0.001 | <0.001 | Yes |
| Regional | US figures, medium skin vs US reference | Chi-square 2x2 vs reference (Yates) | chi2(1) = 116.5 | <0.001 | <0.001 | Yes |
| Regional | US vs Rest of World, medium skin | Chi-square 2x2 (Yates) | chi2(1) = 24.0 | <0.001 | <0.001 | Yes |
| Regional | US vs Rest of World, dark skin | Chi-square 2x2 (Yates) | chi2(1) = 15.7 | <0.001 | <0.001 | Yes |
| Regional | US figures, dark skin vs US reference | Chi-square 2x2 vs reference (Yates) | chi2(1) = 10.8 | 0.001 | 0.002 | Yes |
| Regional | US vs Rest of World, light skin | Chi-square 2x2 (Yates) | chi2(1) = 8.9 | 0.003 | 0.005 | Yes |
| Regional | Rest of World figures, medium skin vs diverse-population reference | Chi-square 2x2 vs reference (Yates) | chi2(1) = 6.6 | 0.010 | 0.016 | Yes |
| Per-country | China, dark skin vs diverse-population reference | Exact binomial | 2/231 (0.9%) | <0.001 | <0.001 | Yes |
| Per-country | South Korea, dark skin vs diverse-population reference | Exact binomial | 1/45 (2.2%) | <0.001 | <0.001 | Yes |
| Per-country | Brazil, dark skin vs diverse-population reference | Exact binomial | 0/21 (0.0%) | <0.001 | <0.001 | Yes |
| Per-country | India, dark skin vs diverse-population reference | Exact binomial | 7/49 (14.3%) | <0.001 | <0.001 | Yes |
| Per-country | India, medium skin vs diverse-population reference | Exact binomial | 31/49 (63.3%) | 0.001 | 0.002 | Yes |
| Per-country | Brazil, medium skin vs diverse-population reference | Exact binomial | 10/21 (47.6%) | 0.509 | 0.554 | No |
| Per-country | China, medium skin vs diverse-population reference | Exact binomial | 95/231 (41.1%) | 0.737 | 0.779 | No |
| Per-country | South Korea, medium skin vs diverse-population reference | Exact binomial | 18/45 (40.0%) | 1.000 | 1.000 | No |
| Temporal | Rest of World figures, dark skin by year (figure-level) | Logistic regression | OR/yr = 1.224, z = 2.35 | 0.019 | 0.029 | Yes |
| Temporal | Rest of World figures, dark skin share by year, 2015-2024 | Pearson correlation (annual %) | r(8) = +0.72 | 0.020 | 0.030 | Yes |
| Temporal | All figures, dark skin by year (figure-level) | Logistic regression | OR/yr = 1.092, z = 1.90 | 0.057 | 0.081 | No |
| Temporal | All figures, light skin by year (figure-level) | Logistic regression | OR/yr = 0.967, z = -1.72 | 0.085 | 0.119 | No |
| Temporal | US figures, light skin by year (figure-level) | Logistic regression | OR/yr = 0.939, z = -1.61 | 0.108 | 0.146 | No |
| Temporal | US figures, light skin share by year, 2015-2024 | Pearson correlation (annual %) | r(8) = -0.44 | 0.198 | 0.257 | No |
| Temporal | Rest of World figures, light skin by year (figure-level) | Logistic regression | OR/yr = 0.971, z = -1.25 | 0.212 | 0.271 | No |
| Temporal | US figures, dark skin share by year, 2015-2024 | Pearson correlation (annual %) | r(8) = +0.42 | 0.223 | 0.279 | No |
| Temporal | US figures, medium skin by year (figure-level) | Logistic regression | OR/yr = 1.050, z = 1.16 | 0.247 | 0.305 | No |
| Temporal | All figures, dark skin share by year, 2015-2024 | Pearson correlation (annual %) | r(8) = +0.40 | 0.257 | 0.311 | No |
| Temporal | US figures, dark skin by year (figure-level) | Logistic regression | OR/yr = 1.073, z = 0.98 | 0.329 | 0.386 | No |
| Temporal | All figures, medium skin by year (figure-level) | Logistic regression | OR/yr = 1.018, z = 0.89 | 0.371 | 0.429 | No |
| Temporal | All figures, light skin share by year, 2015-2024 | Pearson correlation (annual %) | r(8) = -0.30 | 0.396 | 0.451 | No |
| Temporal | Rest of World figures, light skin share by year, 2015-2024 | Pearson correlation (annual %) | r(8) = -0.27 | 0.443 | 0.496 | No |
| Temporal | US figures, medium skin share by year, 2015-2024 | Pearson correlation (annual %) | r(8) = +0.24 | 0.501 | 0.553 | No |
| Temporal | Rest of World figures, medium skin by year (figure-level) | Logistic regression | OR/yr = 1.010, z = 0.43 | 0.668 | 0.717 | No |
| Temporal | Rest of World figures, medium skin share by year, 2015-2024 | Pearson correlation (annual %) | r(8) = +0.09 | 0.802 | 0.835 | No |
| Temporal | All figures, medium skin share by year, 2015-2024 | Pearson correlation (annual %) | r(8) = +0.09 | 0.812 | 0.835 | No |

*Chi-square goodness of fit: observed count vs the count expected from the reference share. Chi-square 2x2 vs reference: observed counts vs reference-expected counts treated as a second sample of equal size, with Yates continuity correction (the test reported in Section 3.4). Exact binomial: two-sided test of k/n against the reference share. Pearson correlation: annual percentage vs year (10 years, df = 8). Logistic regression: figure-level indicator vs calendar year; OR per year with Wald z. Values below 0.001 are shown as <0.001.*
