# Supplementary material for: Skin color representation in orthopedic literature
Source: Arch Orthop Trauma Surg. 2026 Nov 3;146(1):392. doi: 10.1007/s00402-026-06561-4 (PMC13731530; doi:10.1007/s00402-026-06561-4)
Supplement: Supplementary file 3 [file 402_2026_6561_MOESM3_ESM.docx]

*Skin Color Representation in Orthopedic Literature. Archives of Orthopaedic and Trauma Surgery. Electronic supplementary material.*

**Supplementary Table 3. Derivation of the reference distributions**

**Panel A. Fitzpatrick skin phototype by self-reported race, He et al. 2014**

| **Type** | **White** | **Asian** | **Black** | **Native American** | **Latino** | **Multiracial** |
| --- | --- | --- | --- | --- | --- | --- |
| I | 9.5 | 1.4 | 1.4 | 1.2 | 7.9 | 2.0 |
| II | 21.7 | 5.9 | 3.1 | 7.8 | 10.9 | 7.4 |
| III | 29.5 | 13.0 | 6.5 | 8.2 | 18.8 | 18.4 |
| IV | 21.4 | 26.2 | 11.5 | 27.3 | 27.9 | 22.9 |
| V | 12.3 | 33.5 | 29.1 | 35.9 | 22.6 | 32.6 |
| VI | 5.5 | 20.0 | 48.4 | 19.6 | 11.8 | 16.7 |
| n | 1,021 | 576 | 556 | 245 | 340 | 564 |

*Source: He SY, McCulloch CE, Boscardin WJ, Chren M-M, Linos E, Arron ST. Self-reported pigmentary phenotypes and race are significant but incomplete predictors of Fitzpatrick skin phototype in an ethnically diverse population. J Am Acad Dermatol 2014;71:731-7, Table II. Values are percentages within each group, recalculated from the published Table II counts (n row); they may differ from the printed percentages by up to 0.1 point because of rounding. Whole sample (n = 3,386): I 4.6, II 11.1, III 18.1, IV 22.2, V 25.0, VI 18.8%. Phototype in He et al. was self-reported.*

**Panel B. Population weights**

| **Scheme** | **Group or region -> He et al. row** | **Weight (%)** |
| --- | --- | --- |
| US (2020 Census) | White | 57.8 |
| US (2020 Census) | Latino | 18.7 |
| US (2020 Census) | Black | 12.1 |
| US (2020 Census) | Asian | 5.9 |
| US (2020 Census) | Native American | 0.7 |
| US (2020 Census) | Multiracial | 4.8 |
| World (UN WPP 2024, 2023 estimates) | Asia -> Asian | 59.05 |
| World (UN WPP 2024, 2023 estimates) | Africa -> Black | 18.30 |
| World (UN WPP 2024, 2023 estimates) | Europe -> White | 9.21 |
| World (UN WPP 2024, 2023 estimates) | Latin America and the Caribbean -> Latino | 8.14 |
| World (UN WPP 2024, 2023 estimates) | Northern America -> US Census mix (above) | 4.73 |
| World (UN WPP 2024, 2023 estimates) | Oceania -> White | 0.56 |

*US: U.S. Census Bureau, 2020 Census Redistricting Data (Public Law 94-171), Table P2, non-Hispanic single-race shares plus Hispanic or Latino of any race; White 57.8, Hispanic 18.7 and Black 12.1 sum with the remaining groups (Asian 5.9, American Indian and Alaska Native 0.7, multiracial or other 4.8) to 100, the last four matching the published 11.4% for all other groups. World: United Nations, Department of Economic and Social Affairs, Population Division. World Population Prospects 2024; regional populations for reference year 2023 (the latest estimate year, not a projection), obtained via Our World in Data. Shares are of the sum of the six regions. Northern America is weighted by the US Census mix above.*

Mapping world regions onto US racial and ethnic categories is a simplification: He et al.’s Asian, Black, White and Latino respondents were US residents and do not represent the full phenotypic range within Asia, Africa, Europe or Latin America. The world-weighted distribution is therefore an approximation intended to show the direction and rough size of a global reference, not a population estimate.

**Panel C. Resulting three-bin reference distributions (%)**

| **Reference** | **Light (I-II)** | **Medium (III-IV)** | **Dark (V-VI)** |
| --- | --- | --- | --- |
| He et al. whole sample (used in analysis) | 15.7 (16) | 40.3 (40) | 43.8 (44) |
| World-population-weighted | 10.8 | 37.4 | 51.8 |
| US Census-weighted | 23.1 | 44.9 | 32.0 |
| US, Keiser 2012 NHANES (used in analysis) | 34.7 | 52.7 | 12.6 |

*Keiser E, Linos E, Kanzler M, Lee W, Sainani KL, Tang JY. Reliability and prevalence of digital image skin types in the United States: results from National Health and Nutrition Examination Survey 2003-2004. J Am Acad Dermatol 2012;66:163-5, Table I (survey-weighted digital image skin type 1-6: 1.6, 33.1, 47.8, 4.9, 3.6, 9.0%; N = 2,691 adults aged 20-59). The He et al. reference was applied in its rounded form (16/40/44), as in the main analysis.*

**Panel D. Sensitivity of the primary comparisons to the choice of reference (exact binomial p)**

| **Group** | **Bin** | **Observed %** | **He et al. (16/40/44)** | **World-weighted** | **US Census-weighted** | **Keiser 2012** |
| --- | --- | --- | --- | --- | --- | --- |
| Overall | Light | 65.6 | <0.001 | <0.001 | <0.001 | <0.001 |
| Overall | Medium | 29.6 | <0.001 | <0.001 | <0.001 | <0.001 |
| Overall | Dark | 4.8 | <0.001 | <0.001 | <0.001 | <0.001 |
| US | Light | 69.7 | <0.001 | <0.001 | <0.001 | <0.001 |
| US | Medium | 23.3 | <0.001 | <0.001 | <0.001 | <0.001 |
| US | Dark | 7.0 | <0.001 | <0.001 | <0.001 | <0.001 |
| Rest of World | Light | 62.5 | <0.001 | <0.001 | <0.001 | <0.001 |
| Rest of World | Medium | 34.6 | <0.001 | 0.061* | <0.001 | <0.001 |
| Rest of World | Dark | 2.8 | <0.001 | <0.001 | <0.001 | <0.001 |

* Not significant. The single exception is Rest of World medium skin against the world-population-weighted reference (34.6% vs 37.4%, p = 0.061). Every other comparison has the same direction and p < 0.001 under all four references.

Reference-independent threshold: the upper exact (Clopper-Pearson) 95% confidence limits for dark-skin (V-VI) representation are 5.9% overall (n = 2,025), 9.2% for US figures (n = 644) and 4.0% for Rest of World figures (n = 1,092). The overall dark-skin deficit is therefore significant against any reference with more than 5.9% Fitzpatrick V-VI.
