# Supplementary material for: Skin color representation in orthopedic literature
Source: Arch Orthop Trauma Surg. 2026 Nov 3;146(1):392. doi: 10.1007/s00402-026-06561-4 (PMC13731530; doi:10.1007/s00402-026-06561-4)
Supplement: Supplementary file 4 [file 402_2026_6561_MOESM4_ESM.docx]

*Skin Color Representation in Orthopedic Literature. Archives of Orthopaedic and Trauma Surgery. Electronic supplementary material.*

**Supplementary File 1. Classification prompt and model parameters**

**1. Model and request parameters**

| **Item** | **Value** |
| --- | --- |
| Model | OpenAI GPT-4o, requested by API alias "gpt-4o" (no dated snapshot pinned) |
| Endpoint | POST https://api.openai.com/v1/chat/completions |
| Message structure | Single user message: text prompt (below) followed by one image (base64 data URL, JPEG or PNG) |
| Image detail | detail = "high" |
| Output format | response_format = {"type": "json_object"}; max_tokens = 120 |
| Sampling parameters | Not set; API defaults applied (temperature 1.0, top_p 1.0, no seed) |
| Run date | June 26, 2026 (single pass; each figure submitted once) |
| Snapshot served | Not recorded in the original run. In the repeatability rerun (Sept 26, 2026; n = 200) the alias was served by gpt-4o-2024-08-06 |
| Rate limiting | On HTTP 429 the request was retried after the server-specified delay (up to 4 attempts) |
| Response parsing | JSON parsed; Fitzpatrick normalised to I-VI; figure type 1/2/3 mapped to clinical/intraoperative/illustration |

**2. Failed calls (2 of 4,809)**

Two requests returned no usable classification and were not resubmitted. Both were treated as not containing gradable skin and are counted among the 2,784 exclusions in Figure 1:

PMC5146898_Fig1_whole: empty JSON object returned

PMC6320617_Fig5_whole: rate-limit retries exhausted (HTTP 429)

**3. Final prompt (used for 4,748 of 4,809 figures), verbatim**

```
You are a dermatology-aware annotator classifying ONE orthopaedic journal figure. Reply ONLY with a JSON object using these keys:

"skin": "yes" or "no" -- is gradable human skin visible and assessable? Answer "no" for: radiographs/CT/MRI/ultrasound, charts/graphs, gross specimens, or when skin is tiny (<5% of frame), blurred, or fully covered by gloves/drapes/dressings. Answer "yes" when skin is visible even if medical devices (goniometers, rulers, implants, retractors) are also present in the frame.

"type": 1, 2, 3, or null -- ONLY when skin="yes":
  1=clinical photo (skin/limb visible, no open surgical field)
  2=intraoperative photo (open field, retractors, exposed tissue, drapes)
  3=medical illustration (drawn/rendered, not a real photo)
  null when skin="no".

"fitzpatrick": "I","II","III","IV","V","VI", or null. Judge CONSTITUTIVE pigment only -- the intrinsic skin color the person was born with, not the appearance under current lighting. Anchors:
  I  = ivory, pale white, or pale pink; skin that would burn immediately in sun and never tan; in surgical photos this skin often appears almost white or faintly pink
  II = white to light beige; may have a faint warm undertone but remains clearly pale overall
  III = light to medium beige with a warm or olive undertone that is clearly and distinctly different from pale/light skin; tans gradually; assign III only when the skin has an obvious warmth or olive quality beyond what surgical lighting alone would cause
  IV = moderate brown or olive-brown; clearly pigmented at baseline, tan obvious without sun
  V  = dark brown; deep, rich pigmentation
  VI = very dark brown to black; deepest pigmentation
  Use the full I-VI range. Correct for confounds before rating:
  - Betadine/iodine/chlorhexidine stains skin orange-brown -- assess unprepped skin at wound margins instead
  - Surgical lights add warmth and yellow tone to pale skin -- this does NOT indicate Type II or III; judge the underlying pigment
  - Tourniquet pallor, erythema, bruising, jaundice -- not constitutive pigment
  - Blood/hemorrhage does not reflect skin pigment; base rating on unaffected skin visible elsewhere in frame
  null when skin="no".

"confidence": 1-5 or null. Default toward lower scores; only assign 4-5 when criteria are fully met:
  5=unobstructed skin >40% of frame, neutral and even lighting, no prep/blood/pallor, type completely unambiguous; use sparingly
  4=skin clearly visible >20% of frame, minimal prep/blood, one type clearly favored
  3=skin visible but limited by small area, directional lighting, partial prep, or mild blood; assigned type most likely but adjacent type plausible
  2=substantially uncertain: heavy prep, significant blood, blown highlights, deep shadow, or boundary between types; rater disagreement expected
  1=barely gradable: nearly unusable due to lighting, obscuration, or insufficient visible skin
  When between two scores, choose the lower. null when fitzpatrick=null.

Example: {"skin":"yes","type":2,"fitzpatrick":"I","confidence":3}
```

**4. Pilot prompt variant (used for 61 validation figures)**

The 61 validation figures were classified once during prompt testing with a variant that is identical except for the wording of the confidence rubric. Skin, figure-type and Fitzpatrick instructions, anchors and confound corrections are the same. Lines that differ:

*Final prompt:*

5=unobstructed skin >40% of frame, neutral and even lighting, no prep/blood/pallor, type completely unambiguous; use sparingly

4=skin clearly visible >20% of frame, minimal prep/blood, one type clearly favored

3=skin visible but limited by small area, directional lighting, partial prep, or mild blood; assigned type most likely but adjacent type plausible

2=substantially uncertain: heavy prep, significant blood, blown highlights, deep shadow, or boundary between types; rater disagreement expected

1=barely gradable: nearly unusable due to lighting, obscuration, or insufficient visible skin

*Pilot variant:*

5=unobstructed skin >40% of frame, neutral and even lighting, no prep/blood/pallor materially affecting skin, type completely unambiguous with no plausible alternative; use sparingly

4=skin clearly visible and well-lit, >20% of frame, minimal prep/blood, one type clearly favored though one adjacent type cannot be entirely excluded

3=skin visible but limited by at most one of: small visible area, directional lighting, partial prep, mild overexposure, or minor blood; assigned type is most likely but an adjacent type remains a realistic alternative

2=assessable but substantially uncertain: heavy prep, significant blood, blown highlights, deep shadow, pallor, limited skin, or boundary appearance between types; two or more types are reasonably plausible and rater disagreement would be expected

1=barely gradable: skin present but nearly unusable due to lighting, obscuration, image quality, or insufficient visible skin; any assignment is highly speculative

**5. Image filtering code**

The code that determined which of the 40,674 harvested figures were sent for classification is reproduced verbatim below (pipeline files image_utils.py, panels.py, purefilter.py and config.py; Python 3 with NumPy, Pillow and imagehash). The steps were applied in this order: (5.1) every harvested figure or panel was triaged on its RGB pixels; (5.2) near-duplicate images were removed by perceptual hash; (5.3) multi-panel composites were collapsed to the published parent figure; (5.4) the pure-white / pure-black filter was applied to the resulting figure-level images. Exclusions from 5.4 were recorded under the same two reasons used in 5.1, which is how they are grouped in Figure 1.

*5.1 Pixel triage: achromatic imaging and sparse charts (image_utils.py)*

Excluded 9,979 figures as achromatic imaging (at least 92% of pixels with RGB channel spread below 14) and 13,921 as sparse charts or text.

```
_GRAY_DIFF      = 14      # per-pixel channel spread below this = "gray" pixel
_GRAY_FRAC_DROP = 0.92    # >=92% gray pixels -> achromatic -> drop
_WHITE_LEVEL    = 235     # all channels above this = white/background
_WHITE_FRAC_HI  = 0.75    # mostly-white candidate
_INK_FRAC_MIN   = 0.08    # chromatic ink below this on white-heavy img -> chart

def _rgb_small(path: str):
    try:
        im = Image.open(path).convert("RGB")
    except Exception:
        return None
    w, h = im.size
    if max(w, h) > 600:
        s = 600 / max(w, h)
        im = im.resize((max(1, int(w * s)), max(1, int(h * s))))
    return np.asarray(im, dtype=np.int16)

def triage(path: str) -> dict:
    rgb = _rgb_small(path)
    if rgb is None:
        return dict(keep=False, reason="unreadable", photo_likelihood=0.0,
                    gray_frac=None, white_frac=None, ink_frac=None)
    px = rgb.reshape(-1, 3)
    spread = px.max(axis=1) - px.min(axis=1)
    gray_frac = float((spread < _GRAY_DIFF).mean())
    white = px.min(axis=1) > _WHITE_LEVEL
    white_frac = float(white.mean())
    chromatic = (~white) & (spread >= _GRAY_DIFF)
    ink_frac = float(chromatic.mean())

    g, wf, ink = round(gray_frac, 3), round(white_frac, 3), round(ink_frac, 3)
    if gray_frac >= _GRAY_FRAC_DROP:
        reason = ("sparse_chart_or_text" if white_frac >= _WHITE_FRAC_HI
                  else "achromatic_imaging_xray_mri_ct_us")
        return dict(keep=False, reason=reason, photo_likelihood=0.0,
                    gray_frac=g, white_frac=wf, ink_frac=ink)
    if white_frac >= _WHITE_FRAC_HI and ink_frac < _INK_FRAC_MIN:
        return dict(keep=False, reason="sparse_chart_or_text",
                    photo_likelihood=0.05, gray_frac=g, white_frac=wf, ink_frac=ink)
    likelihood = float(np.clip(ink_frac * (1.0 - 0.5 * white_frac) * 2.5, 0, 1))
    return dict(keep=True, reason="kept", photo_likelihood=round(likelihood, 3),
                gray_frac=g, white_frac=wf, ink_frac=ink)
```

*5.2 Duplicate detection (image_utils.py, config.py)*

Excluded 187 duplicate images (perceptual-hash Hamming distance of 4 or less from an image already seen). Images that could not be downloaded (106) or opened (1) were also excluded.

```
PHASH_DUP_DISTANCE      = 4      # perceptual-hash Hamming distance <= this -> duplicate      # config.py

def phash(path: str) -> str | None:
    try:
        return str(imagehash.phash(Image.open(path)))
    except Exception:
        return None

def hamming(h1: str, h2: str) -> int:
    return imagehash.hex_to_hash(h1) - imagehash.hex_to_hash(h2)

def is_duplicate(this_hash: str, seen_hashes: List[str],
                 max_dist: int | None = None) -> bool:
    """True if this_hash is within max_dist of any already-seen hash."""
    if this_hash is None:
        return False
    if max_dist is None:
        max_dist = cfg.PHASH_DUP_DISTANCE
    for h in seen_hashes:
        try:
            if hamming(this_hash, h) <= max_dist:
                return True
        except Exception:
            continue
    return False
```

*5.3 Panel collapse (panels.py)*

Superseded 8,558 sub-panels by their parent composite figure, so each published figure was assessed once.

```
PANEL_SUFFIX = re.compile(r"__panel[A-Za-z0-9]+\.\w+$")

def _original_from_panel(panel_path: str, verify: bool) -> str | None:
    """A kept panel lives in the article dir as <base>__panelX.png; the original
    composite is <base>.<ext> in the same dir. Return it (globbed) or None."""
    base = PANEL_SUFFIX.sub("", panel_path)
    if not verify:
        return base + ".*"            # placeholder for preview/testing
    hits = [h for h in glob.glob(base + ".*") if "__panel" not in os.path.basename(h)]
    return hits[0] if hits else None

def collapse(df, verify=True):
    kept = df[df["auto_decision"] == "keep"]
    changes = {"superseded": 0, "new_whole": 0, "missing_original": 0}
    new_rows = []
    located = {}                       # parent -> original path

    for parent, grp in kept.groupby("parent"):
        panels = grp[grp["panel"] != "whole"]
        if panels.empty:
            continue                   # already a single 'whole' figure; leave it
        rep = panels.iloc[0]
        orig = _original_from_panel(rep["image_path"], verify)
        if verify and not orig:
            changes["missing_original"] += 1
            continue                   # can't find composite -> leave panels as-is
        located[parent] = orig
        row = {c: rep.get(c, "") for c in df.columns if c != "parent"}
        row.update(figure_id=cfg.figure_id(rep["pmcid"], rep["fig_ref"], "whole"),
                   panel="whole", image_path=orig, phash="", is_grayscale="",
                   photo_likelihood="", gray_frac="", white_frac="", ink_frac="",
                   auto_decision="keep", auto_reason="")
        new_rows.append(row)
        changes["new_whole"] += 1

    # supersede the kept panels of every parent we collapsed
    mask = (df["parent"].isin(located)) & (df["panel"] != "whole") & (df["auto_decision"] == "keep")
    changes["superseded"] = int(mask.sum())
    out = df.copy()
    out.loc[mask, ["auto_decision", "auto_reason"]] = ["exclude", "superseded_by_collapse"]
    if new_rows:
        out = pd.concat([out, pd.DataFrame(new_rows)], ignore_index=True)
    return out, changes
```

*5.4 Pure-white / pure-black filter (purefilter.py)*

Applied with pure-white fraction > 0.40, pure-black fraction > 0.50 and channel tolerance 5 (the defaults written in the script are 0.50 / 0.50; the applied values are confirmed by the recorded fractions, the smallest excluded being 0.4005 and 0.5004). Excluded 3,063 figures on pure white, of which 14 were reinstated after the manual audit of borderline cases (net 3,049), and 64 on pure black. Totals in Figure 1: achromatic imaging 9,979 + 64 = 10,043; charts, graphs or text 13,921 + 3,049 = 16,970.

```
MAXDIM = 1200   # nearest-neighbour cap: keeps speed AND preserves exact 0/255 values
WHITE_REASON = "sparse_chart_or_text"              # charts/graphs/text figures
BLACK_REASON = "achromatic_imaging_xray_mri_ct_us"  # X-ray/MRI/CT/US/PET scans

def pure_fracs(path: str, tol: int):
    try:
        im = Image.open(path).convert("RGB")
    except Exception:
        return None, None
    if max(im.size) > MAXDIM:                       # NEAREST so pure pixels survive
        s = MAXDIM / max(im.size)
        im = im.resize((max(1, int(im.size[0]*s)), max(1, int(im.size[1]*s))), Image.NEAREST)
    a = np.asarray(im, dtype=np.int16).reshape(-1, 3)
    white = float(np.all(a >= 255 - tol, axis=1).mean())
    black = float(np.all(a <= tol, axis=1).mean())
    return round(white, 4), round(black, 4)

# decision rule (from main(); a.white = 0.40, a.black = 0.50, a.tol = 5)
rm_white = kept["pw"] > a.white
rm_black = kept["pb"] > a.black
wmask = df.index.isin(rm[rm["pw"] > a.white].index)
bmask = df.index.isin(rm[(rm["pb"] > a.black) & (rm["pw"] <= a.white)].index)
df.loc[wmask, ["auto_decision", "auto_reason"]] = ["exclude", WHITE_REASON]
df.loc[bmask, ["auto_decision", "auto_reason"]] = ["exclude", BLACK_REASON]
```
